# The effect of caricaturing on the esthetic appeal of familiar faces, and its relation to simple proportion judgments

**DOI:** 10.1177/20416695241300099

**Published:** 2024-12-08

**Authors:** Andrew J. Anderson, Margaret S. Livingstone

**Affiliations:** Department of Optometry and Vision Sciences, 85084 The University of Melbourne, Australia; Harvard Medical School, Harvard University, USA

**Keywords:** face perception, caricature, esthetics, spatial vision

## Abstract

It has been suggested that caricaturing enhances esthetic appeal, by making an image more strongly stimulate those areas of the brain encoding the subject's distinctive features than does the subject itself. However, some experimental work suggests that people prefer faces with proportions closer to average, or closer to a particular template. It might be that familiarity with the face is important if caricaturing is to increase the esthetic appeal of a likeness. Here we examined how automated caricaturing of photographs of nominal celebrities influenced judgments of esthetic appeal, and how familiarity with the celebrities affected these. Caricaturing monotonically decreased the esthetic appeal of the celebrity photographs, with subjects’ familiarity with the celebrity not influencing this relationship. The degree to which caricaturing influenced esthetic appeal was not correlated with judgments of relative spatial dimensions for a simple shape, either in a discrimination threshold experiment or a peak-shift experiment.

Ramachandran and Hirstein's (1999) proposed that “all art is caricature,” whereby an essential aspect of something is not only captured by the artist but also exaggerated. Because of this, those parts of the brain that respond to an object's distinguishing features are even more stimulated by the artist's representation of the object. In turn, the viewer's emotional and esthetic response to the artwork is greater than to the object itself. There is evidence that neural responses to faces monotonically increase as facial identity shifts away from average (Leopold et al., 2006; Loffler et al., 2005) and that this response increase continues as a face is caricatured (Leopold et al., 2006). Although Ramachandran and Hirstein sometimes speak about increased esthetic responses neutrally (that is, neither as a positive or negative), their paper's aim was to examine things that “people generally find attractive,” to try to discern a pattern linking these, and assess why this pattern is pleasing. Therefore, here we assume that caricaturing is a device used to increase esthetic appeal.

In contrast to Ramachandran and Hirstein's idea, there is evidence people across different ethnic backgrounds prefer photographic images of faces with proportions approaching the group average (Rhodes et al., 1999). Rhodes and Tremewan (1996) found that caricaturing decreased facial attractiveness in simple line drawings, although the level of caricaturing investigated likely exceeded that used by artists wishing to subtly exaggerate features, rather than make an overt caricature. Moderate and high levels of caricaturing in three-dimensional face models also reduce attractiveness, although such three-dimensional caricaturing linearly increases perceived age (Deffenbacher et al., 1998). In contrast to the idea of preferring average dimensions, the average of highly attractive faces shows systematic deviations from the average of all faces (Perrett et al., 1994; Sofer et al., 2015). This suggests attractiveness might be optimized by deviations in a particular direction. Artistic portraits—when compared to normative facial dimensions—also show deviations in a single direction (e.g., a significant trend for increased eye roundness and decreased lower face roundness) (Costa & Corazza, 2006). A preference for caricaturing would not predict such single direction deviations but rather would predict deviations away from normal regardless of direction (e.g., increased roundness for already above-average roundness eyes, and decreased roundness for already below-average roundness eyes).

Arguments that people prefer proportions closer to average or that deviate in a unitary direction both contrast with Ramachandran and Hirstein's proposal which predicts a preference for greater deviations from average, regardless of direction. It may be that *familiarity* with a face is important. Conformity to some representation of an idealized stereotype—whether it be a population average (Rhodes et al., 1999), or an average representing attractiveness (Perrett et al., 1994; Sofer et al., 2015; Trujillo and Anderson 2023)—may be preferred when faces are unknown (e.g., laboratory photo libraries; prephotographic portraits of historical figures whose true appearance is unknown). Similarly, caricaturing might increase appeal only in a nondirectional way if the original face is already familiar. In other words, caricaturing across any dimension of facial identity—particularly subtle amounts—may only be effective if an observer already is familiar with the uncaricatured proportions of the face and, therefore, the dimension that is being caricatured. A positive association between familiarity and the degree of caricaturing deemed to produce the best photographic likeness of a person has been found previously (Benson & Perrett, 1991). Similarly, the facial recognition advantage for caricatures was lost when faces were relatively unfamiliar (Rhodes & Moody, 1990). However, it might be that the proposal of Ramachandran and Hirstein (1999) is incorrect, and that caricaturing does not increase appeal at all, even if it generates a better likeness (Benson & Perrett, 1991; Rhodes et al., 1987, 1997) that is more easily discriminable (Benson & Perrett, 1991; Rhodes et al., 1997). Even if Ramachandran and Hirstein are correct, it seems reasonable to posit that high levels of caricaturing produce grotesque proportions in photographic images that ultimately reduce appeal. Therefore, here we test the hypotheses that esthetic appeal increases with a small amount of caricaturing (Hypothesis 1) and that the increase in appeal positively correlates with how familiar a face is (Hypothesis 2). Artistic portraits of familiar faces also need to represent the identity of the person, therefore, caricaturing of the sort described by Ramachandran and Hirstein (1999) would need to make a more appealing representation of a *particular* person, rather than make a more appealing face in general. Methods asking people to rate attractiveness generally (Rhodes et al., 1999)—rather than how attractively a particular person has been represented—might not capture this distinction.

The peak-shift effect (Hanson, 1959) is commonly invoked as underpinning the success of caricatures, and it is known that peak-shift effects occur for manipulated faces (at least for recognition tasks (Lewis & Johnston, 1999; Spetch et al., 2004)). In peak-shift experiments, the stimulus most likely to be subsequently judged as matching a training stimulus (S+) is identical to a training stimulus presented in isolation. However, if a negatively rewarded stimulus is added during training (S−), the stimulus most likely to be seen as matching the previously seen training stimulus is now shifted (the peak shift effect) in a direction away from S−. Therefore, an increased matching responses is seen for a more exaggerated version of the training stimulus, along the perceptual dimension separating S+ and S−. The human brain has multiple sensory dimensions along which visual stimuli are processed (Freiwald et al., 2009; Livingstone & Hubel, 1988), and the peak-shift effect appears to be generalizable feature of sensory processing (Gallaghar et al., 2020). It may be that an observer's general susceptibility to exaggerated versions of a training stimulus in peak-shift experiments predicts the degree to which caricaturing shifts an observer's assessment of esthetic appeal. This would be expected if such shifts reflect a generalized process; for example, how readily a person shifts their response criteria when making discriminations. Therefore, here we also performed a simple peak-shift experiment (determining the aspect ratio of a rectangle) to test whether the magnitude of an observer's peak-shift effect is correlated with the degree that caricaturing alters the esthetic appeal of a face.

## Material and Methods

### Equipment

Experiments were developed in Psychopy (Peirce et al., 2019), with stimuli displayed at 1 m on a Cambridge Research Systems Display++ monitor (1920 × 1080 @ 100 Hz; 70 cm wide; mean luminance 60 cd/m^2^) in a dimly illuminated room.

### Participants

We recruited participants through notices placed in a weekly electronic newsletter to staff of the University of Melbourne, as well as via recruitment flyers posted about campus, and through direct invitation. The study complied with the tenets of the Declaration of Helsinki and was approved by the Psychology Health and Applied Sciences Human Ethics Sub-Committee of the University of Melbourne (ID numbers 24183 (pilot study) and 26944 (main experiment)). All participants provided written informed consent before experimentation and were required to have a binocular visual acuity of at least N16 print at the 1 m test distance. All participants declared that they had not taken the University of Melbourne subject “OPTO20004 – Perception, Illusions & Art,” in which the esthetic laws of Ramachandran and Hirstein are discussed in detail.

Thirty-six people (23 female, 13 male), with a median age of 25 (1st to 3rd quartile, 22.3–37.8) participated in our main experiment. Results from our pilot work are also reported in the esthetic preference experimental subcomponent, outlined below. For this pilot experiment, 17 people (8 female, 9 male) with a median age of 43 (29.5–45.5) participated. There was no overlap in the participants for the main and pilot experiments.

### Procedure

Participants in the main experiment performed the following tasks, in order, in a single session lasting approximately 90 min.

#### Width Discrimination

As a measure of a person's absolute ability to judge relative proportions, participants were shown two red (CIE 1931 *x* = 0.647, *y* = 0.342) horizontally oriented rectangles in succession (0.75 s presentation time, with 0.5 s in between) on a mean gray background, and asked to select which was wider, relative to its height. One rectangle had a fixed aspect ratio of 1.5 and was presented either first or second, at random. We adjusted the aspect ratio of the other rectangle by two interleaved staircases that were each initially up/down (4 dB steps) until the first reversal, and then 3-down/1-up (2 dB steps) for four subsequent reversals. An auditory tone sounded for incorrect responses, and the minimum interstimulus interval was 1 s. For each participant, the threshold was the geometric mean of the final four reversals in each staircase. Participants first performed an incomplete practice run of at least 60 s duration to familiarize themselves with the task.

To discourage judgments being made on absolute length, we randomly jittered the overall size of each rectangle on a trial-by-trial basis by between 1 and 1.3× (uniformly distributed), being approximately 3 times width discrimination thresholds (Anderson & Wassnig, 2012). The minimum height of each rectangle was 4.0°. We also randomly jittered the position of each rectangle away from the center of the screen by ±0.4°, both horizontally and vertically. Participants were instructed to ignore the absolute sizes and positions of the rectangles.

#### Peak Shift Effect

Participants were instructed to remember the width, relative to the height, of an initially presented rectangle, and then push the space bar before the disappearance of any subsequently presented rectangle that matched these proportions. The initial rectangle (S+) was 6.8 × 4.0° (*w* × *h*; aspect ratio 1.6) and presented for 10 s. After this, the training phase of the experiment commenced, consisting of 10 presentations (1.75 s presentation time, 0.75 s interstimulus interval) of rectangles with the same aspect ratio as S + and 10 presentations of S− (a rectangle of aspect ratio 1.5), randomly interleaved. A computer-generated voice told participants if they were “correct” or “incorrect” on each training presentation. Subsequently, ten presentations of each of 11 rectangles with aspect ratios of evenly spaced linear steps between 1.1 and 2.1 were presented, but with no auditory feedback. All rectangles were red, and those following the initially presented rectangle had a minimum height of 4.0°, were size jittered by 1 to 1.3± to discourage judgments on absolute width, and were randomly displaced from the center of the screen by ±0.4° horizontally and vertically. Participants were instructed to ignore the absolute size and position of the rectangles. Participants also completed the same experiment, but with the dimensions of S− and S+ swapped, with half of the participants commencing with these swapped stimuli.

#### Esthetic Preference as a Function of Caricaturing Level

We obtained 20 photographs of celebrity faces from the internet (10 male, 10 female). We tried to select images with minimal head tilts, minimal facial shadows that might confuse facial feature registration, no facial hair, apparent European heritage, neutral expressions, and ages approximately matched to those from either the young or middle-aged cohort of the FACES image database (Ebner et al., 2010). We used images taken from between 1950 and 1980, based on image date, or best inference from other information (e.g., the image was publicity for a contemporaneous movie). This was to reduce the likelihood that image features had had their spatial dimensions manipulated, as might occur post the advent of image manipulation tools such as Photoshop. Images were aligned using the automatic face alignment (AFA) toolbox (Gaspar & Garrod, 2021) which uses a Generalized Procrustes Analysis to give an unbiased global alignment of all the detected landmarks, in contrast to normalizing on a single feature (e.g., the distance between the eyes; Galton, 1878). We manually checked to ensure no obvious failures of the AFA toolbox to register features. Areas beyond the image boundary exposed by the alignment process were rendered black. As recommended by the software authors, the jawline was excluded from the alignment process (Gaspar & Garrod, 2021). Faces were processed at a grayscale image size of 578 × 801 (*w* × *h*) pixels, but were displayed in all experiments at 50% of this size (289 × 400 pixels, being 5.8 × 8.0°).

**Figure 1.**
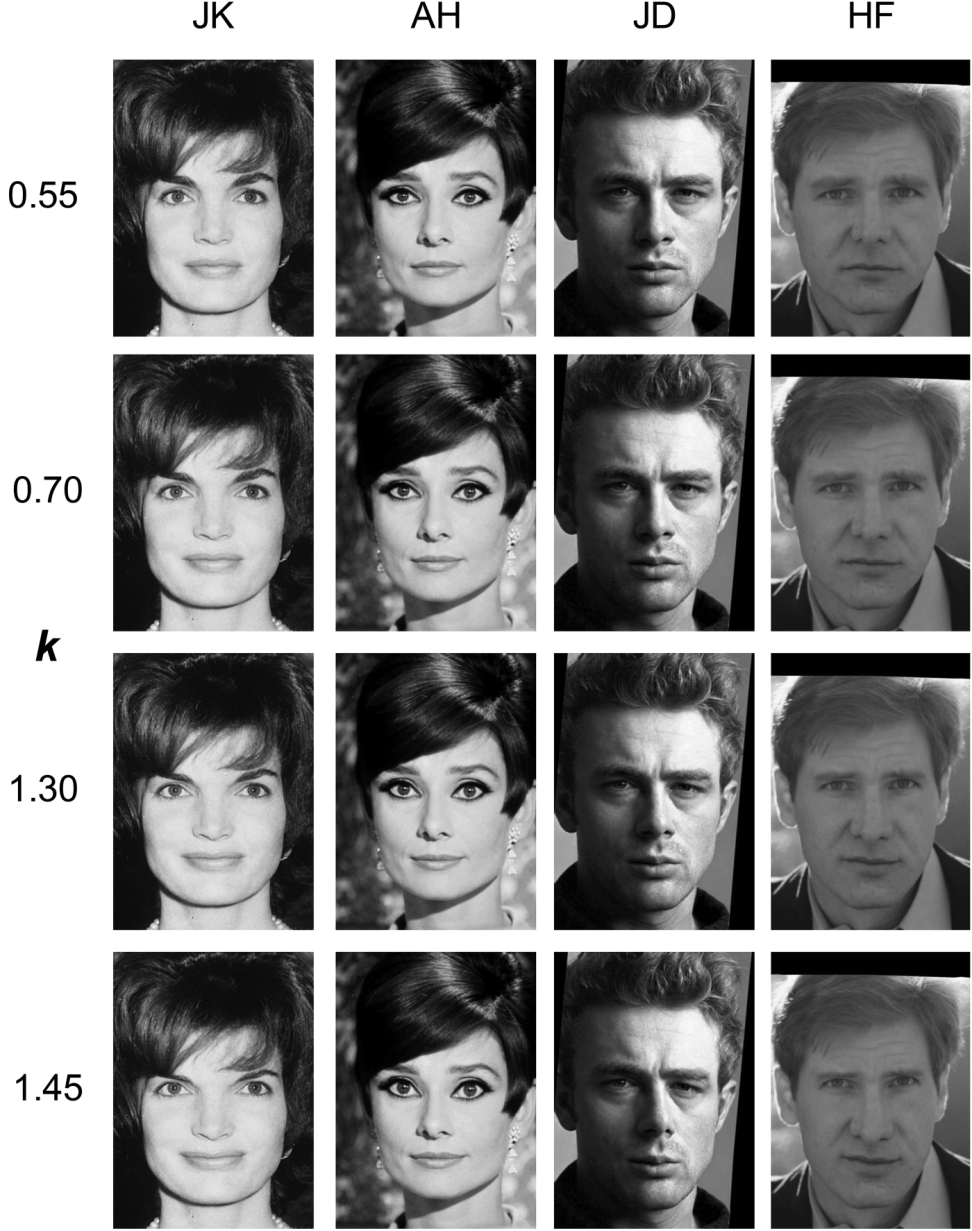
Examples of caricatured (*k *> 1.0) and anticaricatured (*k *< 1.0) celebrity images used in the experiments. The difference between the celebrity's feature location and the average expected location was multiplied by the constant *k*, and images then morphed to have the feature at the location corresponding to this multiplied difference. The average feature locations were taken from the appropriate age- and sex-based subset of the FACES database (young female (JK), middle-aged female (AH), young male (JD), middle-aged male (HF)).

For normalization, we combined the celebrity faces with the appropriate sex (male/female) and age (young/middle-age) images from the neutral “a” subset of the FACES database. These images were then aligned using the AFA toolbox (Gaspar & Garrod, 2021), to create four sets of average facial locations for the combinations for sex and age group. Caricaturing was then applied to our celebrity faces automatically using a modified version of the AFA toolbox function designed to warp a face to an average set of landmarks (Gaspar & Garrod, 2021): here, the altered function warped to an average set of landmarks, plus *k* times the difference between these landmarks and those of the individual face. As such, *k *= 0 warped a face to the average landmarks (=anti-caricaturing), *k *= 1 left the face unchanged, and *k *= 1.5 made a face's landmarks 50% further away from the average location than they originally were (=caricaturing). Values for *k* were 0.55, 0.70, 0.85, 0.95, 1.0 1.08, 1.15, 1.30, and 1.45. Example images are shown in Figure 1.

**Figure 2.**
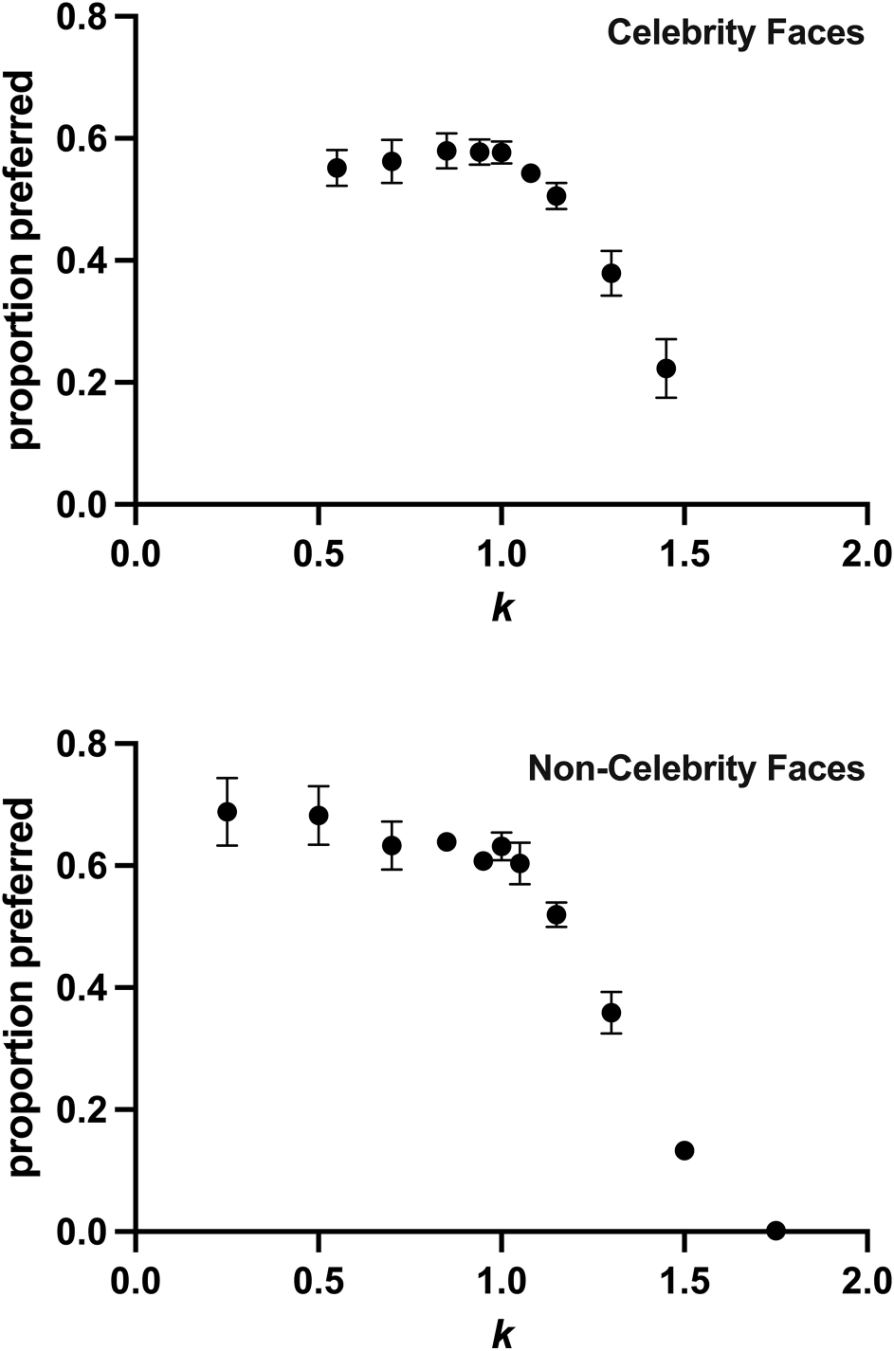
Proportion that an image with a particular *k* was preferred in the forced-choice esthetic preference experiment. Upper panel shows the mean proportion across the 36 observers in the main experiment observing celebrity faces. Lower panel shows the mean proportion across 17 observers in the pilot experiment observing noncelebrity faces. Error bars show the 95% confidence intervals for the mean.

To determine esthetic appeal, horizontally opposed image pairs of the same celebrity were presented, and the participant was forced to choose which was a more appealing portrayal of the person (Anderson et al., 2022; Spehar et al., 2003). We instructed participants to base their judgments on their first impressions, which was aided by the short maximum presentation time of 4 s. This duration exceeds the few hundreds of milliseconds it takes for cortical signals predictive of esthetic choices to emerge (Kaiser & Nyga, 2020), but is likely too quick for significant face adaptation effects (Gao et al., 2022). All nonrepeated pairings for different *k* values were presented, with the left/right ordering randomized, giving a total of 720 image pairs (nine levels of *k* [=36 nonrepeating pairs]×20 celebrities = 720 images). We also included an additional seven presentations each for *k* = 0.70 and *k* = 1.30 (=280 images) compared with the uncaricatured image (*k* = 1.00). These additional presentations were added to our linear mixed effects analysis, described below, but were not included in our calculation of preferences presented in Figure 2. In total, participants judged 1,000 image pairs, which typically took around 40 min. Breaks were enforced when participants had completed 25%, 50%, and 75% of the pairs, with additional breaks available as needed. Images were separated horizontally by 2.2°, with the vertical position of the images randomly jittered vertically ±0.4° each to mask vernier alignment cues to differences between the image pairs. Participants were instructed to ignore the overall size and position of the face.

In addition to this main experiment, a similar experiment was conducted during our pilot experiments, except for a randomly selected young, middle, and older face from the neutral “b” subset FACES database. Caricaturing was relative to the average facial dimensions for either the male or female set of the neutral “b” subset, as appropriate, and faces were Moss-egg windowed using the automatic face alignment toolbox (Gaspar & Garrod, 2021). A wider range of caricaturing values was used (*k *= 0.25, 0.50, 0.70, 0.85, 0.95, 1.0 1.05, 1.15, 1.30, 1.50, and 1.75). All nonrepeated pairings for different caricaturing levels were presented, with the left/right ordering randomized, giving a total of 165 image pairs (55 images for each of the three faces). Participants have familiarity with uncaricatured versions of the faces through searching for neutral “a” versions of the three faces in a visual search task (search for the face among seven other random faces; total 30 exposures per face, across three runs) that preceded the esthetic preference task.

#### Rating Judgments

It is possible that participants might believe caricaturing made an image more artistic, even if it did not increase an image's esthetic appeal. To determine how artistic images were, participants were shown 60 images (*k *= 0.70, 1.0, and 1.3, for the 20 celebrities) in a random order, and asked to rate how artistic they felt each was via a mouse click on a scale from “not artistic at all” (=0) through “of average artistic merit” (=50) to “of the highest possible artistic merit” (=100). The scale and scale definitions were displayed continuously, although faces were shown for a maximum of 2.5 s each.

We also used a rating experiment to determine participants’ familiarities with our celebrity faces. After rating artistic merit, participants were shown the uncaricatured celebrity images in random order and asked to rate how familiar the face would have been to them before commencing the experiment. The scale ranged from “not familiar at all” (=0) to “completely familiar (even if I do not know who the person is, or what they do)” (=1.0). Timing was as per the previous rating experiment.

#### Image Plausibility

On each trial, participants were shown a horizontal sequence of images of a single celebrity, ordered from *k* = 0.94 (left) to *k* = 1.3 (right). Participants were asked to indicate with an appropriate keyboard press the last face that—excluding injury or developmental abnormality—could occur in everyday life (McKone et al., 2014). Participants were also told they had the option to say that all or none of the faces could occur in everyday life. The order of the 20 celebrities was randomized, and image sequences remained on the screen until a selection was made.

#### Demographic Information

At the conclusion of the above experiments, participants were asked their age, sex, interest in visual art on a scale of 1–7 (with the following key provided: 1 = not interested at all; 4 = moderately interested; 7 = extremely interested), approximately how many times they had visited an art gallery or art exhibition in the past three years, and to what degree they considered themselves able to make esthetic choices, on a scale of 1–7 (with the following key provided: 1 = not good at all; 4 = moderately good; 7 = extremely good). An Oxford English Dictionary definition of esthetic (“Of or pertaining to the appreciation or criticism of the beautiful”) was also provided.

#### Detectability of Caricaturing

Seven nonnaïve observers (author AJA, and six others (second-year optometry students) with a detailed knowledge of the study) determined the detectability of the caricaturing levels using a method of constant stimuli. Three horizontally displaced, and vertically jittered, celebrity images were displayed on each trial, and the observer had to select which flanking image did not match the central image. The central image was uncaricatured, with one flanking image also being uncaricatured, and the other being one of the nonunity values of *k* used in the esthetic preference experiment. Images were displayed for a maximum of 6 s, and an auditory tone sounded for incorrect selections. Participants performed 10 repeats, over 10 separate runs. One participant gave proportions correct that decreased monotonically as *k* moved away from 1.0, suggesting they incorrectly interpreted the auditory tone as indicating a correct response: the probability of an incorrect response was therefore analyzed for this participant. An additional naïve participant (a 54-year-old male) also completed a reduced version of the experiment (eight repeats, and with *k* = 0.94 and 1.08 excluded).

### Analyses

A linear mixed effects analysis was performed in R Studio (ver. 2023.09.1 + 494) using the *lme4* package. Our binomial model had the form:

*P(response_ij _= 1) = (A + a_j_) + (B + b_j_) x familiarity_ij _+ c_i_*

where *P(response_ij _= 1)* is the probability, in logit units, of participant *j* preferring the caricatured picture of celebrity *i*, and *familiarity_ij_* is the participant's familiarity rating for the celebrity pictured (from 0 to 1). *A* and *B* are fixed intercepts and slopes of the linear model, respectively, with *a_j_* and *b_j_* random effects (distributed as *N*(0,σ_a_^2^) and *N*(0,σ_b_^2^), respectively) for these coefficients based on participant. A further random effect for the intercept, based on the celebrity being caricatured, is given by *c_i_*. Models were run separately for the *k* = 70 and *k* = 130 conditions, given that we hypothesized a fixed effect of *k* would be nonmonotonic and, therefore, nonlinear. Specifically, moving toward average proportions (*k* < 1.0) has been suggested to increase appeal (see Introduction) and Ramachandran and Hirstein (1999) hypothesize that caricaturing (*k* > 1.0) also increases appeal.

For our peak shift experiment, we determined the location of the matching function peak by a maximum likelihood fit of the following function:

no.ofmatches=FP+(height−FP).N(peak,SD)

where *FP* was the false positive rate, *height* was the height of the function (capped at a value of 10), and *N(peak,SD)* was a normal distribution with a mean of *peak* and a standard deviation *SD*. We specified the size of a person's peak shift as the sum of their individual peak shifts of the parameter *peak* in the S+ and S− training conditions, with a positive shift for each condition meaning a shift away from the negatively rewarded stimulus. The units for the peak shift were the number of steps.

When analyzing our image plausibility data, we analyzed the median value of *k* selected for each celebrity, to allow situations where no image in the series was judged as plausible (i.e., when *k* was not a specific number, but was necessarily less than the smallest value of *k* displayed, i.e., <0.94).

Repeated measures ANOVAs were calculated in Prism 10.1.1 (GraphPad Software, Boston MA), using Geisser–Greenhouse correction.

## Results

The effect of caricaturing in our main experiment can be seen in Figure 2 (upper panel). There was a significant effect of caricaturing level *k* using a one-way repeated measures ANOVA (*F*(2.1, 73.4) = 89.36; *p* < .0001). A Dunnett's multiple comparisons post hoc test showed caricaturing levels ≥1.15 (*p* < .0001) and the anti-caricaturing level of 0.55 (*p* = .014) had significantly different preference rates from the no-caricaturing (*k* = 1.0) condition. Our pilot experiment using noncelebrity images (Figure 2, lower panel) showed similar results, with increasing caricaturing and decreasing preference rates.

**Figure 3.**
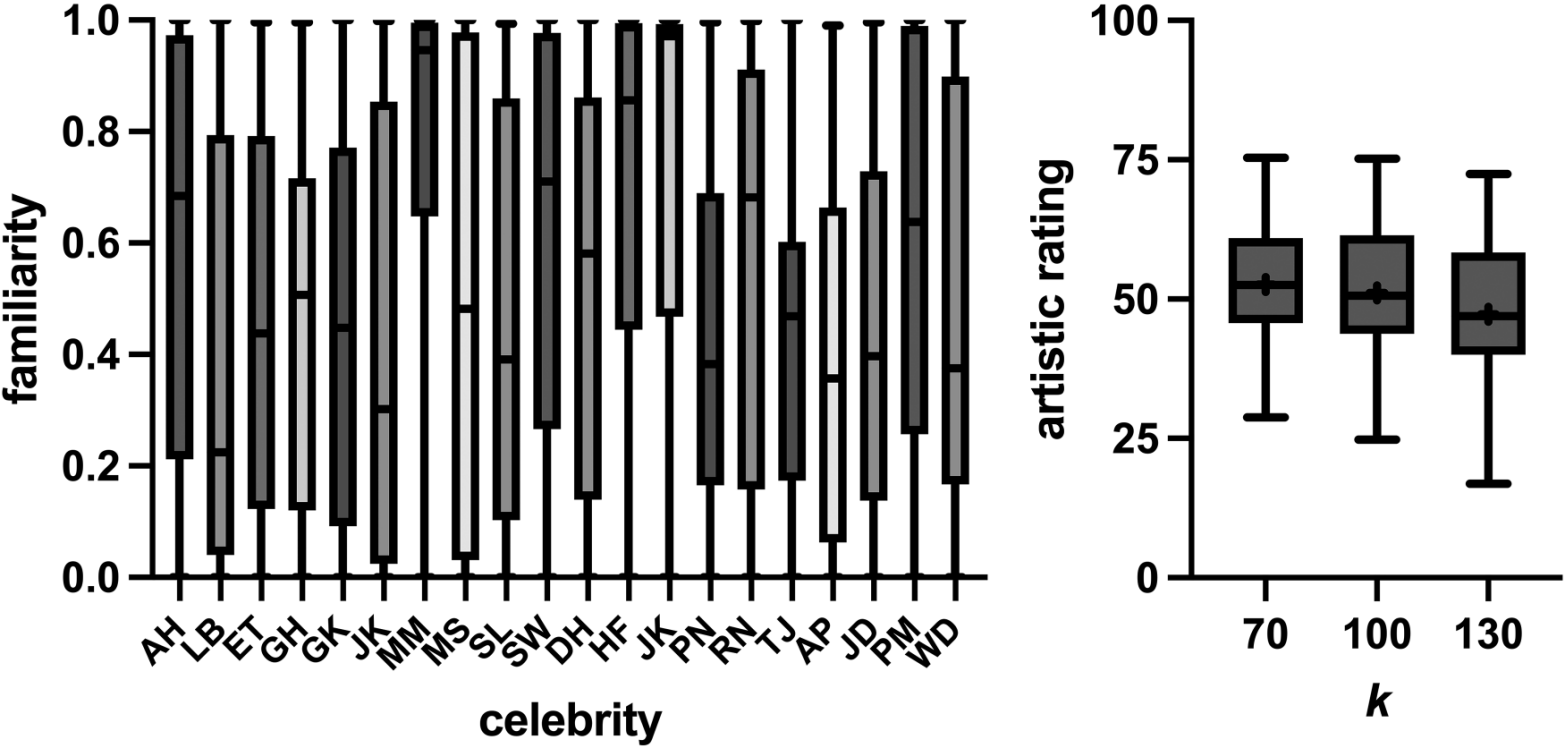
Boxplots showing familiarity ratings for the 20 celebrity images (left panel), along with ratings for how artistic each celebrity image was as a function of *k* (right panel). Whiskers show the maximum and minimum values, and crosses in the right panel give mean values. Celebrity images, as ordered along the *x*-axis, were Audrey Hepburn (AH), Lucille Ball (LB), Elizabeth Taylor (ET), Goldie Hawn (GH), Grace Kelly (GK), Jacqueline Kennedy (JK), Marilyn Munroe (MM), Meryl Streep (MS), Sophia Loren (SL), Sigourney Weaver (SW), Dustin Hoffman (DH), Harrison Ford (HF), John Kennedy (JK), Paul Newman (PN), Richard Nixon (RN), Tom Jones (TJ), Anthony Perkins (AP), James Dean (JD), Paul McCartney (PM), and Willem Daffoe (WD).

For our main experiment, our linear mixed effect modeling showed that participants were significantly less likely to pick the moderately caricatured (*k *= 1.3) image, with a log odds ratio of −0.81 (SE = 0.15, *p* < .0001), indicating that the overall probability of picking this image was 0.31. The fixed effect of familiarity was not significant (log odds = 0.16, SE = 0.11, *p* = .15). Similar modeling for the anticaricaturing condition (*k *= 0.7) showed anticaricaturing did not significantly reduce the probability of picking the image (log odds = 0.03, SE = 0.09, *p* = .74), and there was no significant effect of familiarity (log odds = −0.07, SE = 0.10, *p* = .50). Figure 3 (left panel) shows the median familiarity levels for the celebrity tested, with familiarity ratings ranging from 0 to 1.0 for all images. The median of the range of familiarity levels from each participant was 0.93 (range 0.29–1.0), indicating that our image set represented a range of facial familiarities for each observer, despite all faces notionally being of celebrities.

Figure 3 (right panel) shows how caricaturing influenced how artistic observers believed an image was. There was a significant effect of caricaturing (RM-ANOVA; *F*(1.70,59.5) = 14.06; *p* < .0001), with post hoc testing finding moderate caricaturing (*k *= 130), but not anticaricaturing (*k *= 0.7), decreased the artistic rating relative to the noncaricatured image (*p* = .13 and 0.004, respectively; Dunnett's multiple comparisons test). For our image plausibility experiment, the last image judged plausible had a median *k* of 1.45 (95% CI; 1.15 to 1.45), with a range of 1.08 (2 celebrities) to 1.45 (11 celebrities).

**Figure 4.**
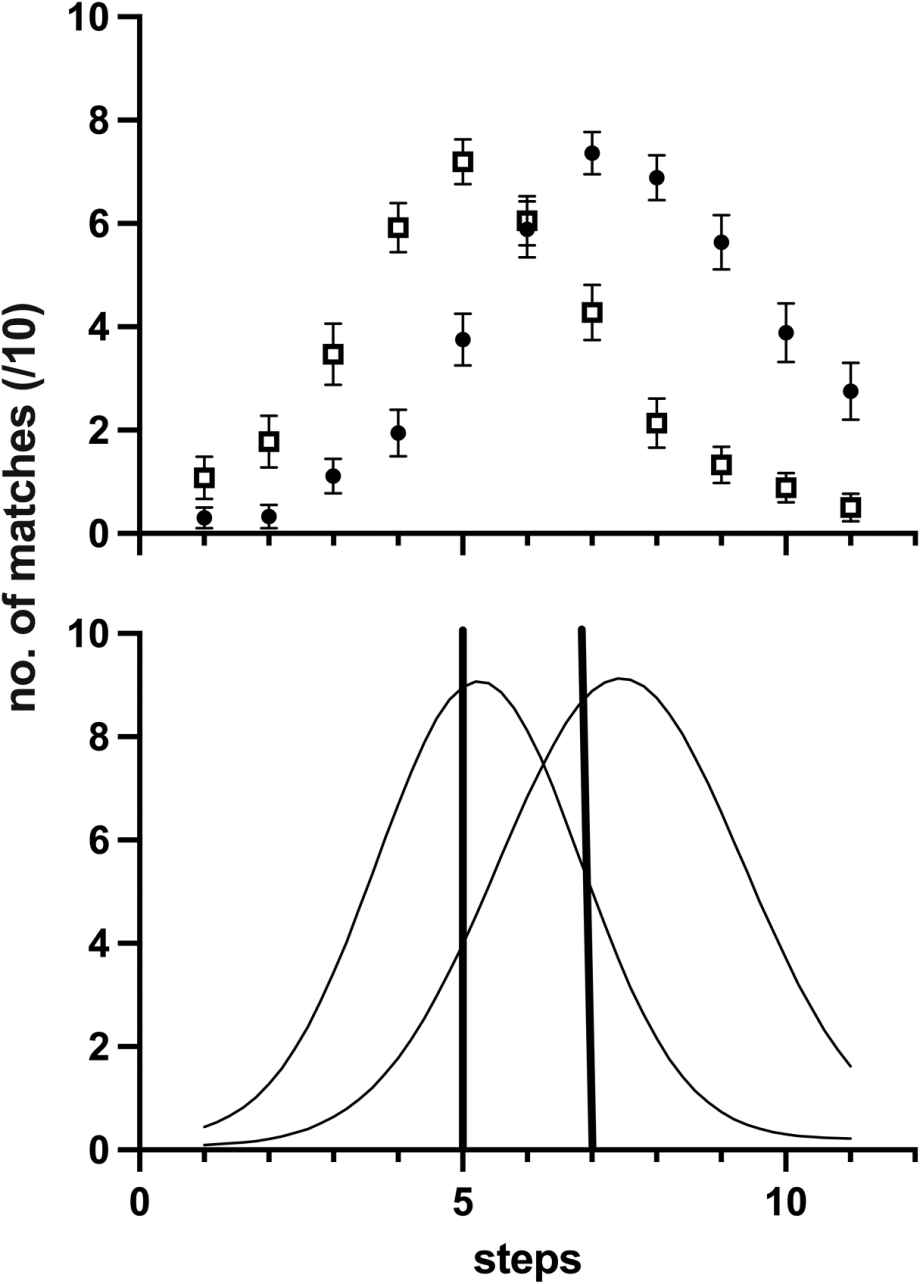
Mean matching data from the peak shift experiment (panel a), and templates generated from the average parameters of the template fits to each participant's data. Steps give linear increases in aspect ratio for the rectangular stimuli, with increasing rectangularity for higher step values. For one experimental run (filled circles), the training stimuli locations (vertical lines, lower panel) were step 7 (S+) and step 5 (S−). For the other run (unfilled squares), the rewarded (S+) and unrewarded (S−) stimuli were reversed.

**Figure 5.**
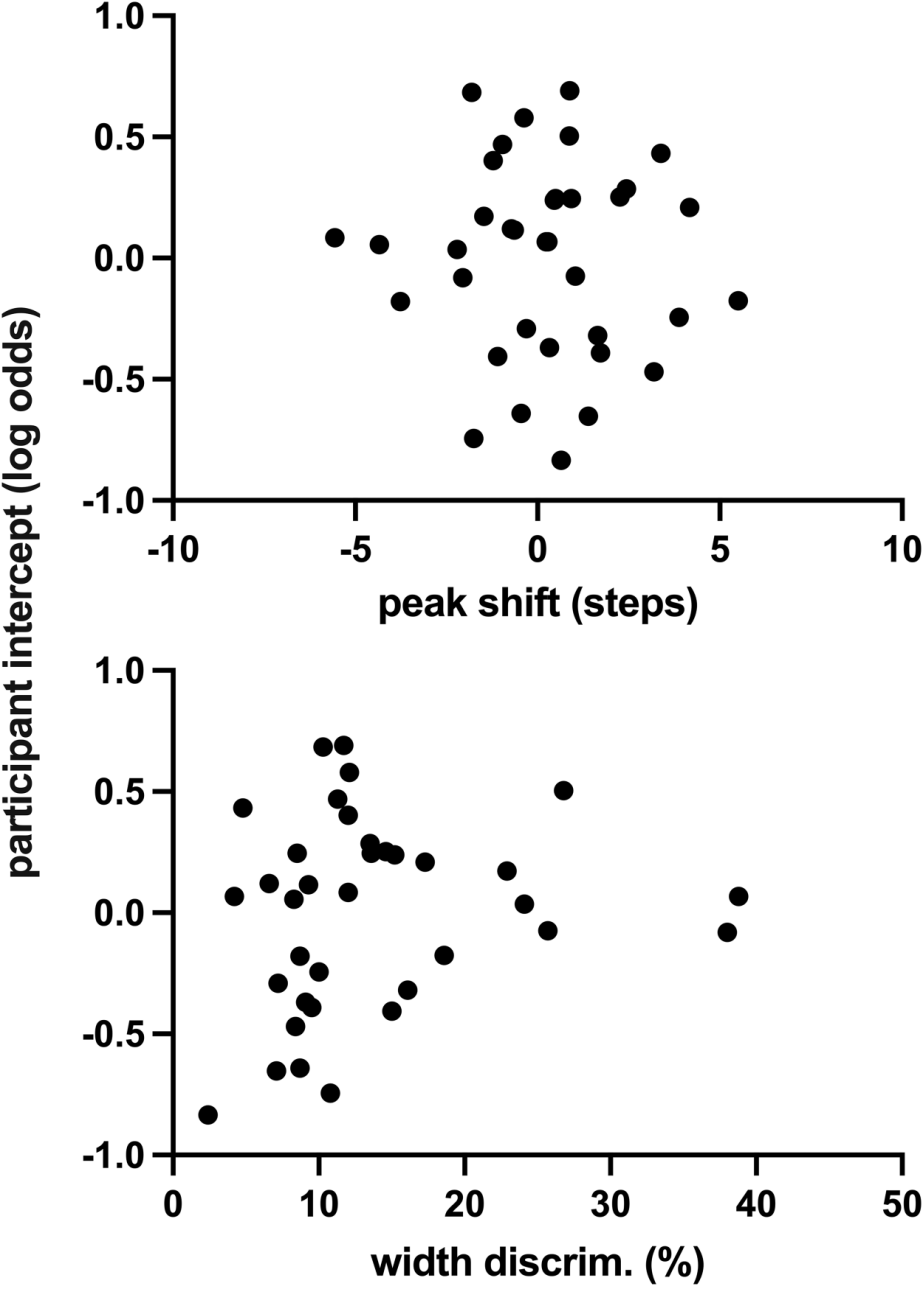
Each participant's random factor intercept for the linear mixed effect model for caricatured (*k *= 130) images, as a function of their peak shift magnitude (upper panel) or their width discrimination threshold (lower panel).

Figure 4 shows the average matching data from our peak shift effect (upper panels), along with matching functions generated from the average parameters of the functions fit to each participant's data. There was a positive peak shift of 0.20 on average, although the confidence interval around this value encompassed zero (95% CI = −0.60 to 0.99). The upper panel of Figure 5 shows each person's peak shift, plotted against their random effect log-odds value in the linear mixed effects model for the *k *= 1.3 condition. The correlation of these data was not significant (Pearson *r* = −0.05, 95% CI −0.38 to 0.28, *p* = .76). Likewise, the correlation between each person's width discrimination and their random effect log-odds value was not significant (lower panel; Pearson *r* = 0.18, 95% CI −0.16 to 0.48, *p* = .29).

**Figure 6.**
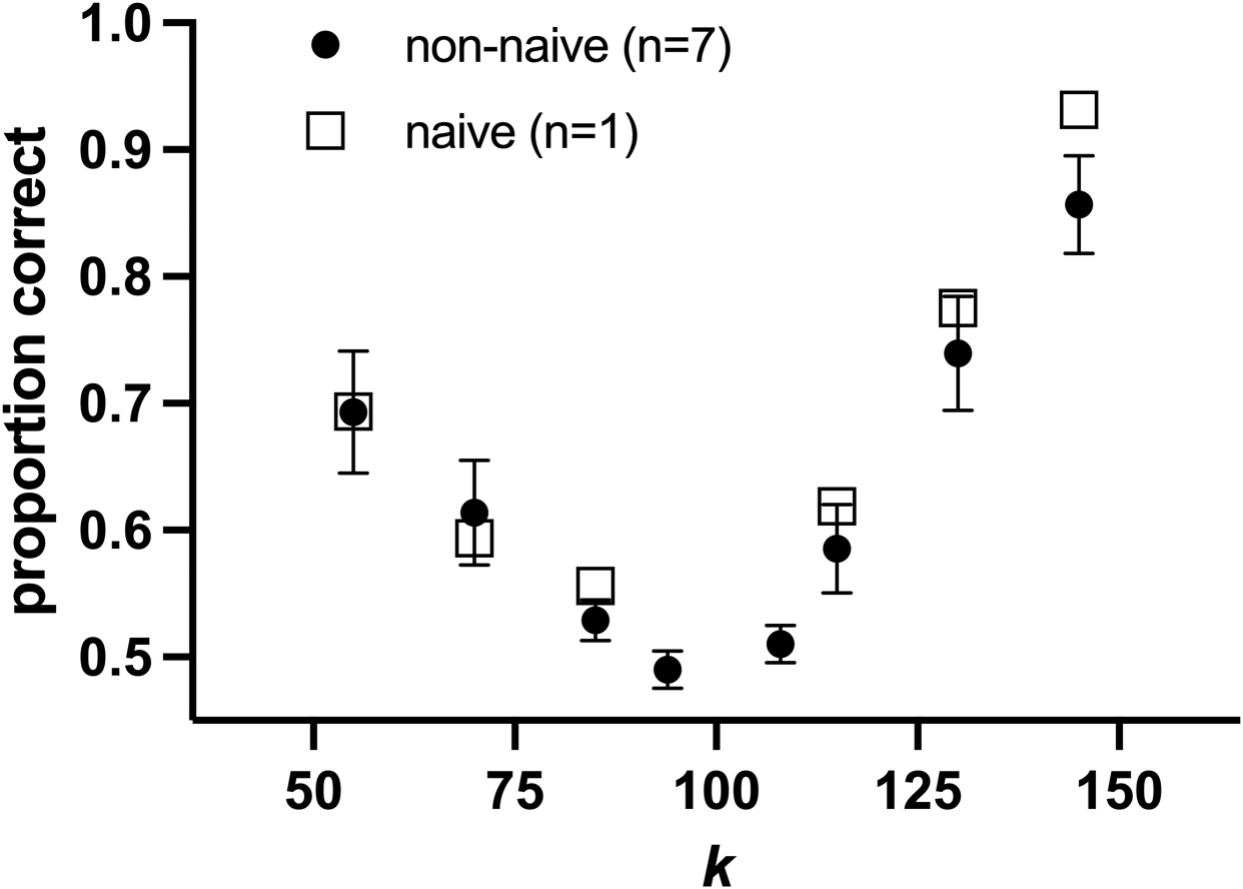
Detectability of the level of caricaturing given by *k*. Filled circles show the mean (±SEM) of six nonnaïve participants, with the unfilled squares showing the results of a single participant naïve to the study's specific aims.

Figure 6 shows how detectable the caricaturing levels were. As *k* moved away from 1.0, the ability to pick this altered image from an unaltered one (*k *= 1.0) monotonically increased. The 95% confidence intervals for all caricaturing levels tested (*k* ≥108), and the anticaricaturing levels ≤0.7, excluded chance performance of 0.5, indicating these images were significantly detectable (*p* < .05). The detectabilities of the *k *= 70 and *k *= 130 conditions, as used in our linear mixed effects analysis, differed significantly (paired *t*-test; *p* = .001).

Participants in our main experiment had a median interest in visual art of 5 (1st to 3rd quartile, 4–6), a median number of 4.5 (2–8.5) gallery visits, and a median esthetic judgment ability of 4.5 (4–5.9). Participants in our pilot experiment had a median interest in visual art of 5 (5–6.5), a median number of 3 (2–6.5) gallery visits, and a median esthetic judgment ability of 4.0 (2.5–5.5).

## Discussion

We found that caricaturing did not increase the esthetic appeal of face images, contrary to the theory of Ramachandran and Hirstein (1999). Our Hypothesis 1 is rejected, therefore, at least for the types of images investigated here. A person's familiarity with a face did not affect the relationship between caricaturing and esthetic appeal, and so our Hypothesis 2 is rejected also. Individual variations in how much caricaturing influenced esthetic appeal were not correlated with two spatial proportion judgments on simple, nonface shapes (the ability to detect differences in aspect ratio, and the susceptibility of aspect ratio judgments to the peak-shift effect). However, the robustness of this latter finding is unclear, given our experiment failed to find a statistically significant peak-shift effect at all.

Our main experiment utilized images of people who were notionally celebrities. This approach successfully created a range of familiarity rankings within each observer, each of whom typically knew some of these celebrities but not others, as expected. However, a potential limitation of this approach is that some people may become celebrities partly due to having above-average attractiveness. Therefore, the monotonic decrease in attractiveness we find with caricaturing (Figure 2, upper panel) could reflect that our celebrity faces were at the upper limit of facial exaggerations related to attractiveness (Perrett et al., 1994). This seems unlikely to us for two reasons. First, at least some people in our images would be celebrities for factors other than attractiveness (e.g., political stature, acting ability, singing ability). Therefore, it seems unlikely that the average of such a group would represent the upper limit of attractiveness compared with a group selected solely on attractiveness. Second, our pilot data using noncelebrity faces (Figure 2, lower panel) showed the same monotonic decrease in appeal with caricaturing.

Both caricaturing and anticaricaturing reduced esthetic appeal. This effect of anticaricaturing is potentially at odds with work suggesting faces with average proportions are more appealing (Rhodes et al., 1999). As noted in the Introduction, it may be that the manipulations toward a template—such as an average—is desirable only for unfamiliar faces whose true facial proportions are therefore unknown. A suggestion of this is seen in our pilot data (Figure 2, lower panel) where noncelebrity faces have increased appeal when anticaricatured. This idea is weakened somewhat given that observers in our pilot experiment did have some familiarity with the faces, having previously located a version of each face in a search task. Furthermore, if familiarity were important, it should be a significant factor in the linear mixed effects analysis of our anticaricaturing data. However, we found no significant effect of anticaricaturing at all at the modest level of *k* (= 0.70), and so there was no effect for familiarity to modulate.

Our data found caricaturing had a larger effect than anticaricaturing (Figure 2). A likely cause of this asymmetry is that anticaricaturing was less detectable than caricaturing (Figure 6). Both *k *= 1.30 and *k *= 0.7 displaced feature locations by equal magnitudes, so one would expect equal detectability if judgments were based on feature location and/or size alone. That this is not so suggests the action of specific face processing mechanisms, where deviations away from average face proportions are more detectable than those toward, at least on average. Dakin and Omigie (2009) found behavioral evidence that the transducer encoding facial identity is nonlinear, which might explain this asymmetry. A direct mathematical prediction of our detection results using Dakin and Omigie's model is not possible, however, given that normalization of the transducer is based on the distinctiveness of particular faces: something not determined in our current experiment. Of note is that the magnitude of a just detectable shift toward average face proportions is larger than just detectable spatial changes in nonface stimuli. For example, changes in spatial frequency (Campbell et al., 1970; Mayer & Kim, 1986) and the separation between line elements (Anderson & Wassnig, 2012) are typically detectable at levels of 10% or less, and the geometric mean of our observers’ width discrimination thresholds in the current experiment (Figure 5) was 12%. In contrast, our observers achieved only 75% detection—a common threshold criterion in 2-AFC experiments—with an anticaricaturing shift around 45% (Figure 6). Whilst this could provide a further indication of a face-specific process, it likely reflects differences in the metrics quantifying change. For conventional spatial metrics, percentages represent changes to the separation between elements (e.g., adjacent bars in a grating). However, in our experiment, percentages represent changes to the difference between an element's position and its reference position (e.g., the difference between this left eye, and the average location for left eyes): the percentage change to the separation between elements in a face (e.g., between right and left eyes) will necessarily be less, therefore.

We used photographic face images in our experiment. Caricaturing might make such images unattractive by making them appear biologically implausible or unrealistic, in a way that paintings—which typically are “implausible” at the outset, in that they would rarely be mistaken for the real object—would not. Therefore, the possibility exists that caricaturing might improve the esthetic appeal of created artworks, even if it does not for photographic representations. Previous work has also found differences between line drawings and photographs, with caricatures enhancing subject recognition in the former only (Rhodes et al., 1997). However, the simple line drawings used in previous studies are poorly recognized in an absolute sense: even with caricaturing, recognition takes around four seconds (Perrett & Benson, 1994). Although it is possible that Ramachandran and Hirstein's thesis holds for a specific subtype of images and manipulations, our current experiment rules out a strong version of their thesis that is robust to the specific characteristics of the images being caricatured. Furthermore, that their thesis might apply to a significant subset of photographic face images seems unlikely, given that our dataset should have contained some of these faces and so increased esthetic appeal on average.

Even accepting that caricaturing might increase esthetic appeal (Ramachandran & Hirstein, 1999), it is reasonable to assume a boundary beyond which this relationship breaks down. Indeed, it has been shown there is a decrease in esthetic preference with large levels of distortion where portraits no longer look like the original (Lewandowski & Pisula-Lewandowska, 2008) or where distortions are beyond natural proportions (McKone et al., 2014). In our current experiment, images were deemed biologically implausible at a median level of *k *= 1.45. Although the fixed caricaturing (*k *= 130) used in our linear mixed effects analysis was less than this, might it still have skipped over an improvement in appeal occurring at even lower caricaturing levels? This seems unlikely, given our Figure 2 shows a monotonically decreasing effect from even at the smallest levels tested (*k* = 1.08), whose detectability is only slightly greater than chance (Figure 6).

Another potential limitation of this study is our caricaturing procedure. Although our approach is common with many studies in the field, the method will not necessarily capture all expected behaviors artists use when caricaturing. Indeed, some automated caricaturing results were judged by a small group of artists to have accentuated the wrong details (Benson & Perrett, 1991). Therefore, it may legitimately be questioned whether the findings of vision research studies, obtained using automated caricaturing, generalize to art practice. In addition, there are likely other facial dimensions (for example, adiposity, race, and age) (Busey, 1998) that could be caricatured but whose spatial characteristics are more complex. Capturing these would necessarily make the caricaturing procedure more computationally complicated and, if performed by artificial intelligence, have the additional complication of the calculation not being explicitly knowable. A further potential complication is that caricaturing by a fixed scale factor may be perceived as having differing effects on different features, as some features may show greater variability than others. Methods to account for this variability have been proposed, however (Mo et al., 2004). Finally, the possibility remains that some artists do employ caricaturing, but for reasons other than increasing esthetic appeal (for example, to increase the distinctiveness of a subject, regardless of how visually pleasing the result).

In summary, our study shows that an automated caricaturing procedure significantly decreased the esthetic appeal of photographic images of both familiar and unfamiliar faces. Our finding stands in contrast to the increase in esthetic appeal with caricaturing predicted by Ramachandran and Hirstein (1999). The possibility that the effect of caricaturing on nonphotographic images—such as painted portraits—is different from what we find here cannot be excluded, although decreased appeal with caricaturing of simple line drawings has been previously found (Rhodes & Tremewan, 1996). How much caricaturing altered a person's esthetic preferences was not related to their visual performance on tasks discerning the proportions of simple geometric shapes.
